# Lower Limb Ischemia: Aortoiliac Thrombosis Related to Antiphospholipid Syndrome (APS)—Case Report and Review of the Literature

**DOI:** 10.1155/2013/536971

**Published:** 2013-07-25

**Authors:** Arnaldo Toffon, Raffaella Piovesan, Consolato Francesco Minniti, Omar Caruso, Paolo Criscenti, Sabina Villalta, Maurizio Cavallo

**Affiliations:** ^1^San Lorenzo Hospital UOSD Vascular Surgery, Valdagno, Italy; ^2^University of Verona, Vascular Surgery Department, Verona, Italy; ^3^Cà Foncello Hospital, Internal Medicine Department, Treviso, Italy

## Abstract

Antiphospholipid syndrome (APS) is recognized as one of the main determinants of hypercoagulable conditions. The literature reports the incidence of this syndrome in a third of patients who underwent surgery for peripheral revascularization. Antiphospholipid antibodies are divided into two categories in relation to specific diagnostic tests. The first group is called lupus anticoagulant and consists of immunoglobulins that inhibit the phospholipid dependent coagulation tests in vitro. The second group is defined by their ability to conduct the phospholipid in an ELISA test. The occurrence of thrombotic events in patients with systemic erythematosus lupus (SEL) and anticoagulant antibodies was described for the first time in 1963 by Bowie. The discovery of anti-cardiolipin antibodies in antiphospholipid syndrome is due to Harris et al. who described the syndrome. Primitive APS was consequently defined in the absence of further underlying illnesses. In this disease, arterial thrombosis occurs mainly in the brain. Peripheral arteries are affected less frequently. Thrombosis of the great vessels is reported as anecdotal.

## 1. Case Report 

**Figure 1:**
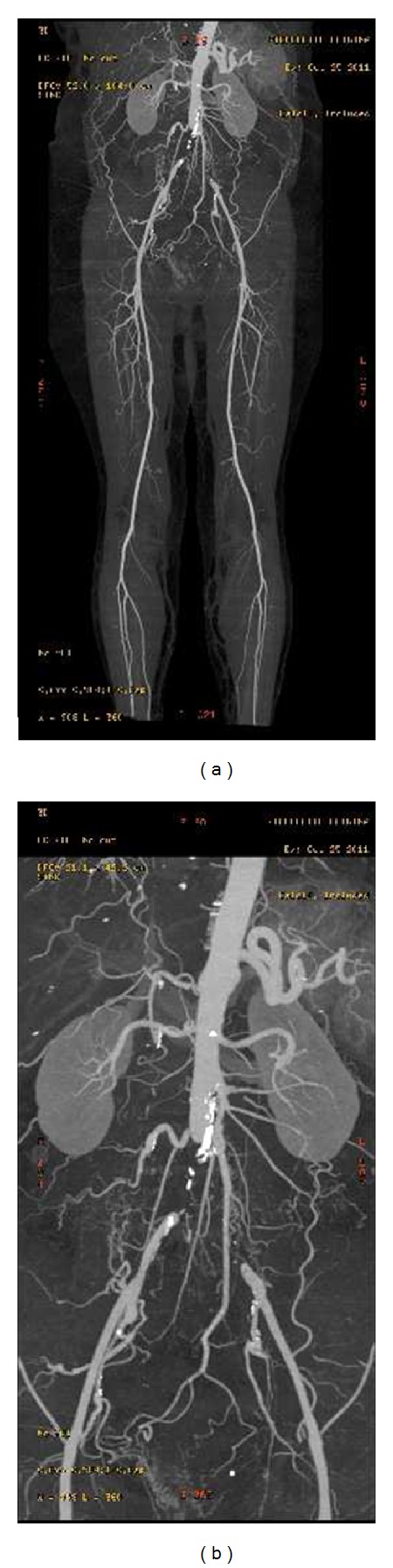
Angio-CT scan images.

Ms. ML, 68 years old, came to our attention after a long diagnostic iter for neurological paresthesias in the lower limbs and suspicious pseudoclaudication. The patient had no history of smoking, hypertension, and diabetes mellitus. The examination detected the absence of femoral pulses. She then performed abdominal ultrasonography and Doppler which showed thrombosis of the subrenal abdominal aorta. She subsequently performed Angio-CT scan (Figures 1(a) and 1(b)), which confirmed the diagnosis of thrombosis of the abdominal aorta and common iliac arteries, while the femoral, popliteal, and tibioperoneal arteries were free from atherosclerotic disease. Preoperative blood examination showed aPTT values of 70.5, aPTT ratio 2.25, and PT (inr): 0.92 (Table 2).

This led us to a more detailed diagnosis by complete screening of blood coagulation. Subsequently we performed screening for lupus antibodies (LA), complementemia, b2 microglobulin, neutrophil cytoplasmic antibodies, microsomal antibodies, DNA antibodies, coagulation factors, and plasmatic RiCoF. These investigations revealed strong compatibility with antiphospholipid syndrome: anti-b2 GP1 IgG: 55.5 U/mL, anti-b2 GP1 IgM: 60 U/mL, anticardiolipin IgG > 150 U/mL, and anticardiolipin IgM: 53.3 U/mL (all values should be <20 U/mL). 

Cardiac ultrasonography showed a mild mitral valve prolapse with mitroaortic insufficiency. Renal function was normal. She presented with hypertriglyceridemia (205 mg/dL) and hypercholesterolemia (total cholesterol: 279 mg/dL with HDL 60 mg/dL). 

She then underwent rheumatological and haematological evaluation. 

The patient was subsequently admitted to hospital and underwent aorto-bisiliac bypass. The examination of the iliac endoluminal material sent to the pathologist highlighted the presence of organized thrombus.

The patient was discharged with anticoagulant therapy (Warfarin) and was advised to maintain INR between 2.5 and 3.3 (Table 3). After one year, the bypass is patent with no evidence of further thrombotic episodes. The values of the Lupus anticoagulant test continue to be positive. 

## 2. Discussion

The majority of thrombotic events occur in the deep venous system of the lower limbs, but they also have been documented elsewhere. Arterial occlusion is described less frequently than the venous occlusions (see Table 1). The most affected areas are the coronary district, the visceral district, the kidney, retina, and peripheral arteries. Aortic disease is a highly unusual event. In fact, in the medical literature, only four cases are reported. Thrombocytopenia is present in about 30% of patients at some stage of evolution of the disease. Other symptoms associated with the disease are livedo reticularis, chronic ulcers, chorea, musculoskeletal events, pulmonary diseases, hypertension, optic neuropathy, and adrenal insufficiency [1].

It should however be remembered that the association between a prothrombotic state and the presence of anticoagulant autoantibodies in vitro is not completely known. In antiphospholipid syndrome, vascular occlusion is due to a thromboembolic event instead of a vasculitis. Some arterial events, however, may also be caused by emboli due to sterile vegetations on heart valves. 

## 3. Diagnostic Difficulty

The antiphospholipid syndrome is clearly a heterogeneous disorder, both in terms of clinical manifestation and for the range of autoantibodies. Due to the risk of thrombosis and the effectiveness of anticoagulant therapy, an accurate diagnosis is conclusive. The diagnosis depends on maintaining a high index of suspicion and confirmation by laboratory investigations [2]. When venous or arterial thrombotic events occur in patients who do not have obvious risk factors, or in which recurrent thrombotic events occur, the condition should be taken into consideration. Although the diagnosis can be performed by evaluating the typical signs and symptoms and laboratory data, they may not be conclusive. There are, in fact, diagnostic and prognostic difficulties due to the presence of antiphospholipid antibodies secondary to infections, related to drugs, and in apparently healthy patients. The first association that has been recognized is antiphospholipid syndrome and syphilis infection. The disease is also manifested in HIV-1, hepatitis C, and other infections, including cytomegalovirus. 

The disease can also be drug induced, and this can lead to confusion in the diagnosis. A proportion of patients treated with chlorpromazine may eventually develop lupus anticoagulant, and patients treated with quinine and quinidine were also involved. Unfortunately, the pathology can be detected in apparently healthy patients. Creagh et al., evaluating 500 women in a state of pregnancy, detected the presence of lupus anticoagulant or anticardiolipin antibodies in 3% of the patients. The identification of such accidental autoantibodies is not associated with an increased risk of arterial thrombosis or abortion, even if a high titer of IgG anticardiolipin has been associated with an increased risk of venous thrombotic disease in an epidemiological study. The heterogeneity of the antibodies in this syndrome requires an approach with laboratory tests. This approach should include coagulation assays and solid phase assays for anticardiolipin; in the case in which both tests are positive, in only 50% of cases, the antiphospholipid syndrome is defined. The persistence of autoantibodies should be assessed over time to exclude transient antibodies that may not have any clinical significance. Unfortunately, there are great limitations with the available laboratory tests. Despite advances in the understanding of lupus anticoagulant and awareness of the limits of coagulation tests, many difficulties remain in the routine diagnostic tests. Lupus anticoagulant with a weak inhibitory activity is often overlooked and deficiencies of coagulation factors are mistaken for lupus anticoagulant [2]. 

In conclusion, the APS is one of the major causes of hypercoagulability in patients with vascular disease. Young age, some vascular risk factors, and the female sex are the presentation features that allow vascular surgeons to suspect the existence of any state of hypercoagulability [1]. 

In our case, the presentation of the disease as aortoiliac thrombosis is associated with few precedents in the literature. We believe that the long-term therapy for patients with this disorder should be anticoagulant, just to avoid the high rate of recurrent thrombosis.

## Figures and Tables

**Table 1:** 

Author	Number	F	M	Age	ART THR	VTE	Obstetric M.
Falcinelli et al. [1]	77	48	29	45 ± 7	30 (39%)	41 (53%)	8%
Atsumi et al. [2]	68	61	7	32	37 (54.4)	31 (45.6%)	39%
Kaushik et al. [3]	42 su 215	na	Na	42 (32–65)	1	21 (50%)	na
Ruffatti et al. [4]	258	223	35	18–65	4	9	53 (%)
Forastiero et al. [5]	120	77	43	40.7	32	45	36.60%
Insko and Haskal [6]	24	15	9	39	17 (70.8%)	7 (29.2%)	13.30%

December 2012.

**Table 2:** 

Characteristics	APL− (*n* = 68)	APL+ (ACL+ or LA+; *n* = 79) (*P* value)	APL+ (ACL+, LA−; *n* = 56) (*P* value)	APL+ (ACL−, LA+; *n* = 12) (*P* value)	APL+ (ACL+, LA+; *n* = 11) (*P* value)
Age (Y)	63	66 (NS)	68 (NS)	61 (NS)	65 (NS)
Mean follow-up period (mo)	35	31 (NS)	31 (NS)	31 (NS)	33 (NS)
Male : female	55% : 45%	45% : 55% (NS)	47% : 53% (NS)	38% : 62% (NS)	27% : 73% (NS)
Heart disease	52%	64% (NS)	69% (.04)	69% (NS)	36% (NS)
Diabetes mellitus	44%	35% (NS)	38% (NS)	31% (NS)	18% (NS)
Hypertension	80%	69% (NS)	76% (NS)	46% (.02)	55% (NS)
Hyperlipidemia	46%	39% (NS)	38% (NS)	46% (NS)	27% (NS)
Smoking history	86%	86% (NS)	87% (NS)	93% (NS)	82% (NS)
Renal failure	20%	17% (NS)	16% (NS)	23% (NS)	9% (NS)
Warfarin therapy	30%	49% (NS)	42% (NS)	54% (NS)	82% (.001)
Suprainguinal procedure	54%	48% (NS)	53% (NS)	39% (NS)	36% (NS)
Infrainguinal procedure	65%	73% (NS)	73% (NS)	77% (NS)	73% (NS)

**Table 3:** 

Type of procedure	APL+ (79 pts)	APL− (68 pts)	*P* value
Aortobifemoral	12	16	NS
Axillofemoral	13	8	NS
Endarterectomy	5	6	NS
Femoral-femoral	6	5	NS
Iliac-femoral	0	2	NS

## References

[1] Falcinelli E, Pompili M, Pengo V, Appolloni V, Guglielmini G, Gresele P (2012). Higher levels of plasma matrix metalloproteinase-2 are associated with a significantly increased risk of arterial thrombosis in patients with the antiphospholipid syndrome. *International Journal of Cardiology*.

[2] Atsumi T, Khamashta MA, Haworth RS (1998). Arterial disease and thrombosis in the antiphospholipid syndrome: a pathogenic role for endothelin 1. *Arthritis and Rheumatism*.

[3] Kaushik S, Federle MP, Schur PH, Krishnan M, Silverman SG, Ros PR (2001). Abdominal thrombotic and ischemic manifestations of the antiphospholipid antibody syndrome: CT findings in 42 patients. *Radiology*.

[4] Ruffatti A, Del Ross T, Ciprian M (2011). Risk factors for a first thrombotic event in antiphospholipid antibody carriers: a prospective multicentre follow-up study. *Annals of the Rheumatic Diseases*.

[5] Forastiero RR, Martinuzzo ME, Cerrato GS, Kordich LC, Carreras LO (1997). Relationship of anti *β*2-glycoprotein I and anti prothrombin antibodies to thrombosis and pregnancy loss in patients with antiphospholipid antibodies. *Thrombosis and Haemostasis*.

[6] Insko EK, Haskal ZJ (1997). Antiphospholipid syndrome: patterns of life-threatening and severe recurrent vascular complications. *Radiology*.

